# Global end-diastolic volume: a better indicator of cardiac preload in patients with septic shock

**DOI:** 10.1186/cc14259

**Published:** 2015-03-16

**Authors:** L Mirea, R Ungureanu, D Pavelescu, I Grintescu

**Affiliations:** 1 Clinical Emergency Hospital of Bucharest, Romania

## Introduction

The aim of the study was to assess the value of the global end-diastolic volume (GEDV) evaluated by transpulmonary thermodilution as an indicator of cardiac preload comparing with stroke volume variation (SVV) in patients with septic shock.

## Methods

A prospective, observational study performed in an interdisciplinary ICU including 91 patients with septic shock. Hemodynamic monitoring was performed with a new calibrated pulse wave analysis method (VolumeView/EV1000; Edwards Lifesciences) in 37 patients (group EV1000) or with an uncalibrated method (FloTrac/ Vigileo; Edwards Lifesciences) in 54 patients (group Vigileo) during the first 72 hours. All patients were receiving mechanical ventilation and vasopressors. Measurements were performed before and immediately after volume loading using 500 ml Ringer solution over a short period (<30 minutes).

## Results

A total of 211 fluid challenges were studied in 91 patients. We observed a significant relationship between the GEDV index before volume loading and the percentage increase in GEDV index in the EV1000 group and changes in GEDV index were significantly correlated with changes in stroke volume index (*r *= 0.75, *P *< 0.001), but an insignificant relationship between SVV variation and cardiac index variation (*P >*0.05) in the Vigileo group.

## Conclusion

The transpulmonary thermodilution GEDV is a better indicator of cardiac preload than SVV in patients with septic shock.

## Acknowledgements

This paper was cofinanced from the European Social Fund, through the Sectorial Operational Programme Human Resources Development 2007-2013, project number POSDRU/159/1.5/S/138907 'Excellence in scientific interdisciplinary research, doctoral and postdoctoral, in the economic, social and medical fields -EXCELIS'; coordinator, The Bucharest University of Economic Studies.
